# The role of peroxisome proliferator-activated receptors in the tumor microenvironment, tumor cell metabolism, and anticancer therapy

**DOI:** 10.3389/fphar.2023.1184794

**Published:** 2023-05-12

**Authors:** Jiaao Sun, Liyan Yu, Xueling Qu, Tao Huang

**Affiliations:** ^1^ Department of Urology, First Affiliated Hospital, Dalian Medical University, Dalian, China; ^2^ Department of Respiratory and Critical Care Medicine, First Affiliated Hospital, Dalian Medical University, Dalian, Liaoning, China; ^3^ Dalian Women and Children’s Medical Center(Group), Dalian, Liaoning, China

**Keywords:** PPARs, cancer, tumor microenvironment, metabolic reprogramming, anti-cancer therapy

## Abstract

Peroxisome proliferator-activated receptors (PPARs) have been extensively studied for over 3 decades and consist of three isotypes, including PPARα, γ, and β/δ, that were originally considered key metabolic regulators controlling energy homeostasis in the body. Cancer has become a leading cause of human mortality worldwide, and the role of peroxisome proliferator-activated receptors in cancer is increasingly being investigated, especially the deep molecular mechanisms and effective cancer therapies. Peroxisome proliferator-activated receptors are an important class of lipid sensors and are involved in the regulation of multiple metabolic pathways and cell fate. They can regulate cancer progression in different tissues by activating endogenous or synthetic compounds. This review emphasizes the significance and knowledge of peroxisome proliferator-activated receptors in the tumor microenvironment, tumor cell metabolism, and anti-cancer treatment by summarizing recent research on peroxisome proliferator-activated receptors. In general, peroxisome proliferator-activated receptors either promote or suppress cancer in different types of tumor microenvironments. The emergence of this difference depends on various factors, including peroxisome proliferator-activated receptor type, cancer type, and tumor stage. Simultaneously, the effect of anti-cancer therapy based on drug-targeted PPARs differs or even opposes among the three peroxisome proliferator-activated receptor homotypes and different cancer types. Therefore, the current status and challenges of the use of peroxisome proliferator-activated receptors agonists and antagonists in cancer treatment are further explored in this review.

## 1 Introduction

Peroxisome proliferator-activated receptors (PPARs) were first recognized as promoters of peroxisome proliferation more than 40 years ago. PPARs are ligand-induced transcription factors that belong to the nuclear receptor superfamily involved in nutrient and energy metabolism, regulating energy homeostasis throughout the body during lipid and carbohydrate metabolism, cell growth, and cancer development (Hong et al., 2019). In 1990, two researchers from the Central Toxicology Laboratory discovered a steroid hormone receptor in mice that is structurally similar to the steroid hormone receptors that have been described previously, but the two receptors are significantly different. The newly discovered receptor can be activated by different molecules, including fatty acids and fibrin, and mediates peroxisome proliferation; it was subsequently named PPARα (NR1C1) and found to be expressed in frogs, mice, rabbits, and humans (Issemann and Green, 1990; Göttlicher et al., 1992; Sher et al., 1993; Guan et al., 1997). Later, in 1992, two more members of the PPARs family, PPARβ/δ (NR1C2) and PPARγ (NR1C3), were discovered in humans and in the *Xenopus* frogs (Dreyer et al., 1992; Schmidt et al., 1992). Since then, due to their role as major regulators of metabolism and body energy homeostasis research on PPARs has grown exponentially. After ligand binding, PPARs bind to peroxisome proliferation reaction elements (PPREs) on DNA and after heterodimerization with retinol X receptors, translocate to the nucleus to regulate the transcription of target genes (Phua et al., 2018; Tan et al., 2021).

All three types of PPARs share the basic structural characteristics of nuclear receptors, including four functional domains: A/B, C, D, and E/F. The A/B domain is an activated functional domain responsible for PPAR phosphorylation. The C domain consists of two zinc fingers and is responsible for PPARs binding to the peroxisome proliferative response element (PPRE) located in the promoter of the target gene. The D domain is the docking site for various cofactors. Finally, the E/F domain is involved in the recruitment of PPARs cofactors during transcription. The E domain is also called the ligand-binding domain, which enables PPARs to bind endogenous or exogenous ligands (Werman et al., 1997; Kota et al., 2005; Heidari et al., 2019). PPARs activated by different ligands participate in different physiological responses, including metabolism and energy homeostasis. The endogenous ligands of PPARs are mostly fatty acids and their derivatives, which are produced by diet, *de novo* synthesis of fatty acids, and lipolysis (Woller et al., 2016). The three PPARs subtypes are highly homologous but encoded by different genes, distributed in different tissues, and exhibit different behavioral patterns in biological functions (Berger and Moller, 2002; Bensinger and Tontonoz, 2008; Schupp and Lazar, 2010). PPARα is mainly expressed in the liver, brown adipose tissue, the heart, kidney, and muscle tissue, is involved in β-oxidation and fatty acid transport, and regulates lipid balance. PPARβ/δ is commonly expressed in skeletal muscle, adipose tissue, the heart, and the gastrointestinal tract and is involved in fatty acid metabolism. PPARγ is expressed in adipose tissue and immune cells and is mainly responsible for regulating adipocyte differentiation and improving insulin resistance (Gross et al., 2017; Corrales et al., 2018). Based on this, PPARs are used to treat different aspects of the metabolic syndrome. Before PPARs were discovered, fibrin, a PPARα agonist, was used as a lipid-lowering drug and remains the mainstream treatment for atherosclerotic dyslipidemia and atherosclerosis (Jellinger et al., 2017). The clinical effects of PPARs are not limited to metabolic disorders. So far, PPAR agonists have been tested in many human diseases, including neurodegenerative, psychiatric, autoimmune, inflammatory and malignant diseases, with varying degrees of success (Hong et al., 2018; Cheng et al., 2019).

Previous studies have shown that PPARβ/δ activation is associated with tumor progression, whereas PPARα and PPARγ activation is associated with tumor suppression. The role of PPAR in cancer has gradually become a research hot spot because such a generalization seems inappropriate due to the complex regulatory signals of PPARs, and their deep mechanism remains to be explored in detail (Cheng et al., 2019). Most anti-cancer therapies target cancer cells and largely ignore the tumor microenvironment (TME) component. The TME or tumor stromal community, consists of non-malignant host cells and non-cellular components. Over the past few decades, the role of TME in cancer progression and therapeutic efficacy has become apparent, and the function of PPARs in these stromal cells has received increasing attention and affects cancer progression directly and indirectly. The cellular environment of cancer cells is composed of homogeneous cell clusters along with highly dynamic and heterogeneous communities of distinct cell types, including fibroblasts, adipocytes, immune, endothelial, inflammatory, and mesenchymal stem cells, which are collectively termed tumor stromal cells (Balkwill et al., 2012). Cancer development is a complex and dynamic process involving three stages: initiation, progression, and metastasis, and the interaction between tumor stromal cells and cancer cells is critical for each step of tumorigenesis, during which cancer cells exhibit plasticity and resistance to various stressors and physiological signals (Kadosh et al., 2020). At the same time, the metabolism and bioenergetics of cancer cells are very different from those of normal epithelial cells, and the high basal metabolic rate and abnormal neovascularization in the TME provide cancer cells with greater capacity for self-consumption of energy. The surrounding stromal cells may also play an essential role in this process.

We reviewed the mechanism of action of three subtypes of PPARs in cancer initiation and development, especially the TME energy metabolism and tumor cell mutations, along with the current progress and challenges of targeted PPARs in cancer treatment, to clarify the molecular basis and development direction for early cancer control.

## 2 The role of PPARs in TME

### 2.1 PPARs in tumor cells

In hepatocellular carcinoma, ectopic expression of PPARα in cancer cells significantly inhibits cell proliferation and induces apoptosis. Specifically, the antitumor function of PPARα is mediated by NF-κB, as manifested by inhibition of NF-κB promoter activity, decreased levels of p65, p50, and BCL2, and increased levels of IκBα protein (Zhang et al., 2014). The deletion of PPARα in the intestine increases the expression of DNMT1 and PRMT6, which in turn reduce the expression of tumor suppressor genes Cdkn1a (P27) and Cdkn3b (p2) through DNA methylation and histone H1R21 methylation-mediated transcriptional inhibition, respectively, to further promote colon carcinogenesis (Luo et al., 2019).

In addition, most studies support the anti-cancer effects of PPARγ; for instance, a 2019 study found that high expression of PPARγ was associated with a good prognosis in patients with colorectal cancer (Yaghoubizadeh et al., 2020). PPARγ inhibits colorectal cancer by regulating cell differentiation and the expression of cell cycle regulators (Chen et al., 2005; Drori et al., 2005). In cervical and liver cancer, PPARγ upregulates the expression of the tumor suppressor gene PTEN, suppresses the PI3K signaling pathway, and reduces the self-renewal and aggressiveness of cancer stem cells (Liu et al., 2013a; Bigoni-Ordóñez et al., 2018). Regarding cancer cell proliferation, multiple cyclins and cyclin-dependent kinases (CDKs) are positive regulators of cell cycle progression, and activated PPARγ promotes cyclin D1 ablation and induces cell arrest (Motomura et al., 2000; Huang et al., 2005). Similarly, when PPARγ is activated by ligand binding, it interacts with Sp1 to stimulate p21 gene transcription and thereby induces the G0/G1 phase arrest in human colorectal and gastric cancer cells (Liu et al., 2018). In another *in vivo* study in a rat model of breast cancer, direct upregulation of PTPRF gene expression by PPARγ had some inhibitory effect on tumor cell proliferation (Xu et al., 2019).

Recently, the dual roles of PPARβ/δ in cancer have been fully explored, most of which are cancer-promoting activities, especially in colorectal cancer (Wagner and Wagner, 2020). First, the IL-6/STAT3 pathway is a key signaling pathway in colitis-associated colorectal cancer. In mouse models of colitis-associated colon cancer, PPARβ/δ increased IL-6 expression and phosphorylation of STAT3, promoting tumorigenesis, while the concomitant 15-lipoxygenase-1 in colon epithelial cells inhibited these effects by downregulating PPARβ/δ (Mao et al., 2015). In mouse models of skin cancer, ultraviolet light exposure induced PPARβ/δ activity, further stimulated Src expression, increased Src kinase activity, enhanced the EGFR/Erk1/2 signaling pathway, increased EMT markers expression, and increased tumor burden (Montagner et al., 2014). In prostate cancer cells, PPARβ/δ, as a key target of transforming growth factor β1 (TGF-β1), activates the cholesterol transporters ABCA1 and cave protein-1 (Cav1), leading TGF-β1 to induce tumor growth, migration, and invasion (Her et al., 2013).

**FIGURE 1:**
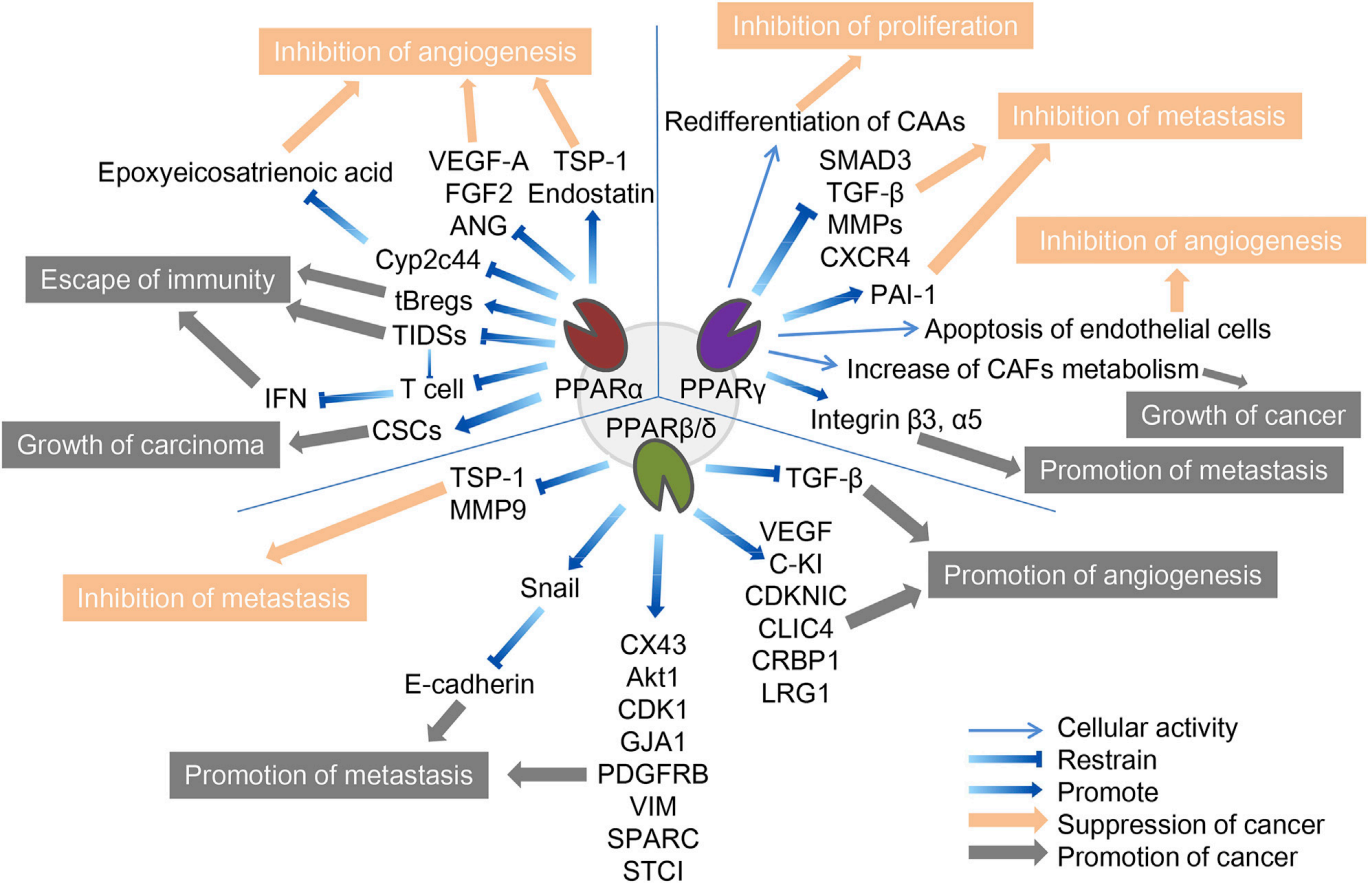
The role of three PPAR subtypes in TME. The three PPARs in TME both promote and inhibit cancer. PPARα inhibits cancer by inhibiting angiogenesis and promotes cancer through escape from immunity and growth of the carcinoma. PPARγ inhibits cancer by inhibiting f proliferation, metastasis and angiogenesis, and promote cancer by promoting tumor growth and metastasis. PPARβ/δ inhibits cancer by inhibiting metastasis and promotes cancer through promotion of metastasis and angiogenesis. The orange arrows and boxes indicate inhibition of cancer, and the gray arrows and boxes indicate promotion of cancer. The detailed mechanisms of action are indicated by blue arrows, including up- and downregulation of cellular activities in which certain cytokines or PPARs are involved.

We believe that the different environment of tumor cells may cause differences in the function of the three PPAR subtypes on cancer cells. Therefore, we will further analyze the mechanism of PPARs in TME (Figure 1).

### 2.2 The role of PPARα in TME

Since tumor cells grow rapidly and require adequate nutrients and oxygen supply, they enhance local blood perfusion by releasing angiogenic factors that would otherwise lead to hypoxia and death, a process significantly influenced by PPARα, which can inhibit angiogenesis to varying degrees (Eales et al., 2016). It is important to mention that functional tumor endothelial cells (TEC), which evolved from endothelial cells lining the vascular system, contribute to the progression of most cancers by supporting tumor metabolism, secreting paracrine factors, and suppressing anti-tumor immune responses (Yang et al., 2021). First, the synthetic PPARα agonists fenofibrate and Wy-14643 have been shown to inhibit vascular endothelial cell proliferation and tumor xenograft growth, whereas mice with PPARα deficiency showed a notable (*p* < 0.05) increase in neovascularization (Pozzi et al., 2007; Panigrahy et al., 2008). Mechanistically, PPARα affects vascular endothelial growth factor (VEGF) and fibroblast growth factor-mediated endothelial cell proliferation and migration by increasing antiangiogenic factors (thromboreagin-1 and endostatin) and decreasing pro-angiogenic factors (VEGF-A and angiopoietin) (Arima et al., 2017). In addition, NOX1 of the NADPH oxidase (NOX) family is a key mediator of angiogenesis, and PPAR-α is also a downstream regulator of NOX1-mediated angiogenesis, whose activity is inhibited by the presence of NOX1, indicating that in NOX1-deficient cells, the upregulated expression of PPAR-α blocks the angiogenic signals required for endothelial cell migration, germination, and angiogenesis (Garrido-Urbani et al., 2011). Finally, in endothelial cells, PPARα reduced epoxyeicosatrienoic acid production through inhibition of arachidonic acid cyclooxygenase expression and also inhibited angiogenesis (Pozzi et al., 2010).

Unlike its role in angiogenesis, PPARα′s effect on immune cells in TME is often pessimistic, ultimately leading to immunosuppression or even cancer immune escape. Tumor-infiltrating dendritic cells (TIDCs) are essential for the regulation of anti-tumor immunity, presentation of tumor-associated antigens to effector T cells, and induction of memory T cells to limit tumor cell growth and recurrence (Diamond et al., 2011). TIDCs that migrate to regional lymph nodes initiate antitumor T cell responses, where they are able to cross antigens from phagocytes to naive CD8+T lymphocytes (Hargadon, 2020). Tumor-derived exosomes carrying fatty acids significantly activate PPARα in TIDCs, resulting in increased intracellular lipid content and mitochondrial respiration, driving immune dysfunction and cytotoxic T-cell activation. In addition, colon cancer grew more slowly in PPARα knockout mice than in wild-type mice, and TIDCs from PPARα knockout mice showed stronger anti-tumor effects than wild-type mice, suggesting that the PPARα signaling pathway is crucial in the immune function of TIDCs, and is involved in DC cell dysfunction caused by TME (Yin et al., 2020). Similarly, in the biological functions of T cells, the expression of PPARα is downregulated after T cell activation, and the activated PPARα can antagonize the F-κB signaling pathway and cytokine production in lymphocytes, indicating that PPARα is an endogenous inhibitor of T cell activation (Jones et al., 2002). IFN-γ levels produced by T cells were higher in PPARα knockout mice than in wild-type mice. The androgens in human T cells increased PPARα expression, resulting in decreased IFN-α and increased IL-17 production in male CD4^+^ T cells (Dreyer et al., 1992; Zhang et al., 2012). Compared to T cells, the role of PPARα in B cells has not been well studied. It has been reported that lipid metabolite of leukotriene B4 produced by breast cancer cells acts as an endogenous PPARα agonist and induces immunosuppressive regulation of B cells (tBregs), thereby promoting distant metastasis of cancer cells. Inhibition of PPARα in B cells can block the production of tBregs, suggesting that targeting PPARα may be beneficial in the regulation of tBregs-mediated cancer escape (Wejksza et al., 2013). PPARα has a similar cancer-promoting effect in cancer stem cells. Cancer stem cells (CSCs) are the most self-renewing subpopulation in the TME, and their activity is positively correlated with malignant tumor progression; the higher the number of CSCs, the greater the potential for tumor development. By activating the stearoyl-CoA desaturase 1 pathway, the activated PPARα was significantly upregulated for the maintenance of CSC stemness in human liver cancer (Ma et al., 2019). 4-Phenylbutyric acid (4-PBA) is a low molecular weight fatty acid that does not induce long-term liver tumors by itself. However, 4-PBA can promote the occurrence of hepatocellular carcinoma by activating the β-catenin signaling pathway to regulate PPAR-α for the initiation of liver cancer stem cells (Chen et al., 2021a).

### 2.3 Role of PPARγ in TME

In addition to being important components of the TME, stromal cells such as tumor-associated fibroblasts (CAFs) and tumor-associated adipocytes (CAAs) provide tumor cells with essential nutrients, including glutamine, L-lactic acid, fatty acids, and ketone bodies. As an important component of tumor stromal cells, CAFs affect the activation of immune cells in TME and the deposition of extracellular matrix (ECM), and its activation is often a key feature of malignant tumors (Chen et al., 2021b; Zheng et al., 2023). These catabolic capacities are mostly secondary to ROS-induced stress responses, mechanistically mediated by HIF1-α and NF-κB signaling. Among them, PPARγ regulates several metabolic remodeling processes in CAFs. With the activation of NFκB and the significant upregulation of COX-2 expression, PPARγ expression was significantly upregulated in CAFs of cutaneous squamous cell carcinoma and colorectal adenocarcinoma (Vandoros et al., 2006; Chan et al., 2018). Increased glycolysis and L-lactic acid secretion in CAFs overexpressing PPARγ was also observed, while the growth rate of MDAMD-231 breast cancer cells was significantly accelerated by the implantation of such CAFs (Avena et al., 2013). In addition, under hypoxic conditions, PPAR γ-dependent hypoxia-inducible factor 1α (HIF-1α) exacerbated the autophagic phenotype of tumor stromal cells, and the HIF1α-PPARγ-UCP2-AMPK pathway significantly affected the mitochondrial biological function of CAFs, resulting in the metabolic reprogramming of CAFs and exacerbating breast cancer growth (Boutoual et al., 2018; Wang et al., 2019). Therefore, PPAR γ turned CAFs into an energy-exporting machine to support tumor growth.

In addition to CAFs, mesenchymal stem cells can differentiate into adipose precursor cells, which further differentiate into CAA after stimulation by PPARγ, which is called adipogenic differentiation. Normal fat cells are mainly considered energy reservoirs to store surplus fuel, and new evidence suggests that they are also important endocrine cells, capable of producing a variety of hormones and chemokines that influence tumor behavior (Wolins et al., 2006; Deng and Scherer, 2010). In pancreatic cancer, cancer cells initiate de-differentiation of adjacent CAAs while the number of CAFs increases, accompanied by the loss of fat cell markers such as leptin, HSL, and PPARγ, and the increase of fibroblast markers such as matrix metalloproteinase (MMP)11 and α-SMA, which further drives the progression of pancreatic tumors (Zoico et al., 2016; Cai et al., 2019). In a mouse model of lip sarcoma, the activation of Notch signaling weakened PPARγ ligand activity, which induced dedifferentiation of CAAs and aggravated tumor-like manifestations, while PPARγ agonists effectively promoted adipocyte redifferentiation and delayed tumor progression (Bi et al., 2016). Similarly, in breast cancer, the deletion of specific PPARγ in CAA downregulates BRCA1 expression and accelerates tumor formation and progression (Skelhorne-Gross et al., 2012). In breast cancer cells *in vitro* and *in vivo*, the activation of PPARγ induces cancer cells to differentiate into fat cells and induces apoptosis of fat cells through the upregulation of transcription factor C/Ebpβ, thereby inhibiting breast cancer cell growth (Li et al., 2015).

Like PPARα, PPARγ exhibits antiangiogenic effects. Previous studies have shown that ligand-activated PPARγ can inhibit human umbilical vein endothelial cell (HUVEC) tube formation and VEGF-induced choroidal neovascularization *in vitro* and *in vivo*, and the ligand 15d-PGJ2 of PPARγ has been shown to directly induce endothelial apoptosis (Bishop-Bailey and Hla, 1999; Xin et al., 1999).

However, contrary to the CAFs-dependent pro-tumor properties, PPARγ reduces hepatoma cell metastasis by inhibiting the transcriptional activity of MMPs and smad family member 3 (SMAD3), thereby reducing hepatoma cell metastasis (Reka et al., 2010; Shen et al., 2012). Activation of PPAR-γ inhibits transforming growth factor β (TGF-β)-induced EMT in lung cancer cells and prevents metastasis by antagonizing SMAD3 function (Reka et al., 2010). Plasminogen activator inhibitor-1 (PAI-1), a member of the serine protease inhibitor family of serpins, inactivates urokinase-type plasminogen activators and inhibits extracellular matrix degradation. There is increasing evidence that PAI-1 is involved in cell migration, tumor invasion, and metastasis. In hepatocellular carcinoma, β-estradiol (E2) activates PPARγ in cancer cells, and activated PPARγ inhibits cell invasion by upregulating the expression of PAI-1 (Pang et al., 2013). By inhibiting NF-κB, activated PPARγ significantly reduced the expression of pro-inflammatory, pro-angiogenic, and pro-transfer signaling molecules in the TME, including IL-6, IL-8, CXCR4, MMP2, and MMP9, which further inhibited the activity of tumor cells in breast cancer (Papi et al., 2014; Rovito et al., 2016). But at the same time, TAM, as one of the most abundant immune cell groups in TME, may contribute to carcinogenesis by mediating neovascularization, immunosuppression and chemical resistance (Wang et al., 2021). Activation of PPARγ in macrophages leads to lipid retention and PGE2 secretion, favoring their polarization toward anti-inflammatory tumor-associated macrophages (TAM), reducing M1 macrophage biomarkers, and tilting towards the M2 phenotype, thereby altering macrophage fate and reducing the Stat3-mediated pro-inflammatory response (Penas et al., 2015; Souza-Moreira et al., 2019; Gionfriddo et al., 2020; Christofides et al., 2021). The decrease of such proinflammatory macrophages reduces the host’s defenses against microorganisms and weakens anti-tumor immunity (Strack et al., 2020). However, in melanoma, activation of PPARγ enhances the expression of surface integrins, specifically integrin β-3 and integrin α-5, increasing the capacity for distant metastasis and implantation of cancer cells, a process also associated with the inhibition of thioredoxin-interacting protein (Meylan et al., 2021). Therefore, the role of PPARγ on TME may be diverse, and further research is needed to explore its deeper mechanism.

### 2.4 Role of PPARβ/δ in TME

Unlike normal fibroblasts, CAFs are paracrine to tumor cells and disrupt the extracellular matrix, often exacerbating cancer spread. However, CAFs-selective PPARβ/δ deficient skin cancer mice showed some remission of metastasis. Similar results were later observed in mouse models of colon cancer (Yoshida et al., 2019; Tan et al., 2020). Mechanistically, the PPARβ/δ knockout CAFs significantly increased the production of reactive oxygen species (ROS) in the neighboring cells, and subsequently activated the RAF/MEK-mediated NRF2, which induced a strong cytoprotective response. Subsequently, the activated NRF2 reduced the phosphorylation of many oncogenes and upregulated the expression of the tumor suppressor gene PTEN. At the same time, it reduced the oncogenic activity of Src and Akt, thereby delaying the tumor process (Tan et al., 2018). In melanoma cell lines with high metastatic potential, activation of PPARβ/δ causes upregulation of Snail and increased sensitivity to signaling stimuli for migration and invasion, where Snail acts as a transcription factor to inhibit E-cadherin transcription in epithelial cells, a phenomenon that can be reversed by inhibition of PPARβ/δ (Ham et al., 2014). In colon tumor tissues, the overexpression of PPARβ/δ intensified the activation of the adhesion protein β-catenin and several factors involved in cancer cell invasion, such as connexin 43, platelet-derived growth factor receptor β (PDGFRβ), Akt1, EIF4G1, and CDK1, all of which promote colorectal cancer progression (Liu et al., 2019a). Similarly, PPARβ/δ also regulates novel metastasis genes such as GJA1, VIM, SPARC, STC1, and SNCG, accelerating the aggressiveness of colon cancer cells (Zuo et al., 2017).

In contrast, the synthetic ligand GW501516 decreased migration and invasion capacity in breast cancer cell lines *in vitro* following activation of PPARβ/δ. Its mechanism is thought to be regulated by platelet-reactive protein-1 (TSP-1) and its degrading proteases, and activated PPARδ significantly inhibited breast cancer cell migration and TSP-1 expression (Ham et al., 2017). Similarly, after GW501516 activated PPARβ/δ, the expression of MMP9 was downregulated, and the invasion ability of pancreatic cancer cells *in vitro* decreased (Coleman et al., 2013).

Contrary to the inhibition of neovascularization by PPARα and PPARγ, PPARβ/δ is a nuclear receptor that promotes angiogenesis. As discovered in 2006, PPARβ/δ accelerates endothelial cell proliferation and enhances tumor cell feeding and metastasis by increasing VEGF, PDGFR, and c-KI biosynthesis (Piqueras et al., 2007; Wagner et al., 2019). Furthermore, the expression of other potential angiogenic mediators including cyclin-dependent kinase inhibitor 1C, IL-8, intracellular chloride channel protein 4, and cellular retinol-binding protein 1 are also affected by PPARβ/δ (Adamkiewicz et al., 2007; Müller-Brüsselbach et al., 2007; Meissner et al., 2010). Leucine-2 glycoprotein 1 (LRG1) is a unique protein produced by tumor tissue, and its inhibition has been shown to normalize blood vessels in tumors (O'Connor et al., 2021). Transforming growth factor β (TGF-β) is a multifunctional cytokine of the transforming growth factor superfamily, that upregulates tumor suppressor genes, induces differentiation, and improves cellular antioxidant properties (Meng et al., 2016). PPARβ/δ upregulates LRG1 expression in CAFs while attenuating the response of epithelial cells to TGF-β (Sng et al., 2018). In patients with colorectal and pancreatic cancer, LRG1 levels tends to be positively correlated with more advanced cancer stages and worse prognosis, and the PPARβ/δ agonist, GW501516, significantly increases LRG1 expression, strongly indicating that LRG1 is a direct target of PPARβ/δ (Zhou et al., 2017; Liu et al., 2019b; Xie et al., 2019). Compared with normal lung tissue, the expression of PPARβ/δ, Cox-2, c-PLA, PGES, and VEGF was increased in human non-small cell lung cancer, and tumor progression was associated with upregulation of PPARβ/δ, increased VEGF levels, and activation of the PI3K/Akt pathway. Specifically, VEGF induction is due to PPARβ/δ binding to the VEGF promoter, and PI3K/Akt pathway activation is due to PPARβ/δ interaction with the PI3K regulatory subunit p85α, resulting in PI3K activation and Akt phosphorylation (Genini et al., 2012).

PPARβ/δ plays a unique role in combating endothelial apoptosis. As early as 2001, prostacyclin was found to induce apoptosis by activating PPARβ/δ in HEK293 cell lines, while endothelial cells in the cytoplasm expressing prostacyclin receptors are not affected by apoptosis. From this, prostacyclin-mediated activation of PPARβ/δ is seen to promote vascular endothelial apoptosis in cells lacking prostacyclin receptors (Hatae et al., 2001). A subsequent study further demonstrated that PPARβ/δ exerts anti-apoptotic effects and promotes angiogenesis by activating endothelial cell 14-3-3ε protein (Brunelli et al., 2007). Prostacyclin 165,041 and carbonaceous prostacyclin (cPGL2) have a protective effect on H2O2-induced apoptosis, and both substances can increase the expression of PPARβ/δ (Liou et al., 2006). There is also evidence that the axis of the COX-2/PPARβ/δ signaling pathway may be important in the development of colorectal cancer by promoting angiogenesis (Yoshinaga et al., 2009). A recent study of the downstream activation targets of PPARβ/δ in angiogenesis demonstrated that blood vessel density increased and tumor growth and metastasis were enhanced in animals with vascular-specific overexpression of PPARβ/δ. Further RNA sequencing was carried out to identify the downstream targets of PPARβ/δ as PDGFRβ), platelet-derived growth factor subunit B (PDGFb), and tyrosinase KIT (c-Kit) (Wagner et al., 2019).

In conclusion, the three types of PPARs play vital roles in cancer tissues, affecting the synthesis of neovascularization, the activity of cancer-like stem cells, the secretion of stromal cells, and the anti-tumor immune process. In such a complex regulatory network, it is impossible to conclude that a certain type of PPAR can inhibit or promote a certain type of cancer; rather, they often form a mutual regulatory cycle.

## 3 PPARs in the energy metabolism of cancer cells

PPARs play a key role in regulating multiple metabolic pathways such as glucose homeostasis, lipoprotein metabolism, fat production, and fatty acid uptake, and the dysregulation of these metabolic processes can lead to the onset of certain metabolic diseases, such as diabetes, nonalcoholic fatty liver disease, and atherosclerosis. In a fasting state, PPARα accelerates the formation of fatty acids by lipolysis in hepatic adipose tissue by regulating the expression of apolipoprotein, increases the level of plasma high-density lipoprotein cholesterol, and decreases the level of low-density lipoprotein cholesterol (Berthou et al., 1996; Schoonjans et al., 1996; Vu-Dac et al., 2003). PPARγ decreased the content of free fatty acids in all organs except adipose tissue and circulating blood, thereby increasing the triglyceride storage capacity of adipose tissue (Gross et al., 2017). In contrast, the effects of PPARβ/δ on different nutrient supply states were not significantly different. PPARβ/δ can promote the catabolism of fatty acids in skeletal muscle and inhibit lipogenesis in adipose tissue (Derosa et al., 2018). PPARα antagonizes the role of insulin in glucose homeostasis, promotes glycolysis and new fatty acid synthesis, and reduces gluconeogenesis, whereas PPARγ plays opposite roles in glucose homeostasis, including increased insulin sensitization in skeletal muscle, improved glucose-stimulated insulin secretion in pancreatic beta cells, and increased gluconeogenesis in the liver, and PPARβ/δ is important in promoting glycolysis, glycogen storage, and reducing gluconeogenesis (Finck et al., 2005; Lee et al., 2006; Ahmadian et al., 2013).

**FIGURE 2:**
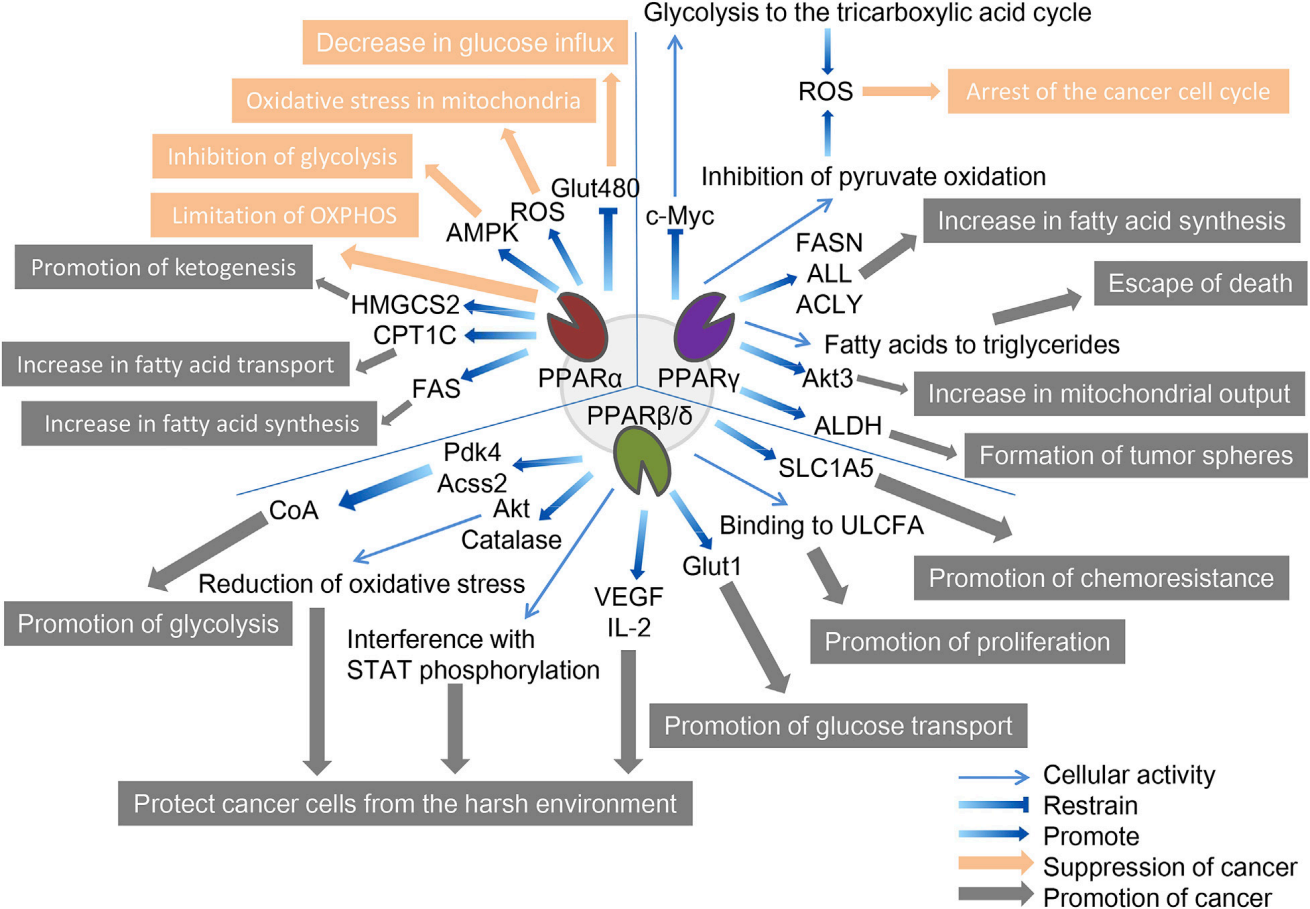
The role of three PPAR subtypes in tumor cell metabolism. All three PPAR subtypes are extensively involved in the special energy metabolism of tumor cells. Compared with the other two PPARs, PPPARβ/δ showed a strong tumor-promoting effect. PPARα inhibits tumors through decreasing glucose influx, oxidative stress in mitochondria, inhibition of glycolysis, and limitation of OXPHOS, and promotes tumors through promotion of ketogenesis, increase in fatty acid transport, and increase in fatty acid synthesis. PPARγ inhibits tumors through the arrest of the cancer cell cycle and promotes tumors through the increase in fatty acid synthesis, escape from death, increase in mitochondrial output, and formation of tumor spheres. PPARβ/δ promotes tumors through promotion of glycolysis, protecting cancer cells from the harsh environment, promotion of glucose transport, promotion of proliferation, and promotion of chemoresistance. The orange arrows and boxes indicate inhibition of cancer, and the gray arrows and boxes indicate promotion of cancer. The blue arrows indicate effector factors involved in regulating metabolism.

Metabolic reprogramming is essential for cancer cells to adapt to changing environments as tumors develop the features of aggressive cancers. In TME, tumor cells exhibit more active metabolic behavior, making their growth possible in this harsh environment. Among these features, increasing glucose uptake and glycolysis, called the Warburg effect, is the first well-known adaptive metabolic event, providing the most direct source of energy for cancer cells, which is essentially glycolysis in the oxygen microenvironment; enhanced glutamine depletion and dissolution provide carbon and amino nitrogen; abnormal lipid metabolism compensates cancer cells for glucose-based energy and biofilm components and regulates proliferation, survival, and metastasis (Altman et al., 2016; Hay, 2016; Luo et al., 2017). While metabolic disorders of cancer cells may directly affect cancer progression, there is early evidence that the activation of PPARs affects tumor metabolism by interfering with the Warburg effect, and the mechanism of action of three PPARs subtypes in cancer cell metabolism will be explored (Grabacka et al., 2013; Han et al., 2017a; Han et al., 2017b) (Figure 2).

### 3.1 PPARα

Activation of PPARα regulates gene expression of specific proteins involved in mitochondrial and peroxisomal function, which dominate the β-oxidation of fatty acids, glucose metabolism, and fatty acid transport (Feige et al., 2006; Rakhshandehroo et al., 2010). The genes regulated by PPARα can determine their cancer-promoting or cancer-suppressing effects due to their relationship with tumor metabolism; for example, inhibitory and promoting effects have been reported in melanoma and breast cancer, respectively (Suchanek et al., 2002; Grabacka et al., 2006).

In terms of cancer promotion, CSCs, as the starting cell population of tumor tissue, exhibit similar self-renewal and differentiation characteristics to normal stem cells and significant upregulation of PPARα, and are important in energy metabolism (Fidoamore et al., 2016). Glioblastoma stem cells are metabolically reprogrammed under hypoxic conditions by upregulating glucose transporters, glucose uptake, and glycogen and lipid storage, in a process that results from the activation of PPARα by hypoxia-inducible factor-1 (HIF-1). The use of GW6471 to antagonize PPARα activity was found to improve glucose and lipid tumor metabolism, thereby reducing glioblastoma proliferation (Fidoamore et al., 2017). In another study, two renal cell cancer cell lines (Caki-1 and 786-O cell lines) were cultured using GW6471, and higher levels of PPARα were observed in high-grade cancer cells than in low-grade cancer cells, requiring high levels of fatty acid oxidation due to their need for more energy, which is regulated upstream by PPARα signaling (Perroud et al., 2009). Furthermore, blocking PPARα in renal cell cancer cell lines under the synergistic effect of glycolytic inhibition significantly reduced the levels of the cell cycle-related proteins cyclin D1 and CDK4, and blocked the cell cycle in the G0/G1 phase, thereby reducing cell viability (Shah et al., 2007; Wang et al., 2011). Fatty acid synthase (FAS) is significantly upregulated in urinary tumors, the metabolic intermediate in the process of fatty acid synthesis is the endogenous ligand of PPARα. Activated PPARα further regulates glucose, lipid, and cholesterol metabolism of tumor cells, and inhibition of FAS in mice can lead to PPARα dysfunction. In addition, PPARα induces fatty acid synthesis by upregulating FAS, and increased FAS expression may indicate tumor aggressiveness and poor prognosis in renal cell carcinoma (Chakravarthy et al., 2005; Chakravarthy et al., 2007; Horiguchi et al., 2008). Elevated expression of genes involved in fatty acid oxidation and glucose metabolism have also been observed in human hepatocellular carcinoma tissues, including PPARα, carnitine palmitoyl transferase 1A (CPT1A, an FAO rate-limiting enzyme), glyceraldehyde 3-phosphate dehydrogenase (G3PDH), and cyclin D1 (Kurokawa et al., 2011). Similar to CPT1A, carnitine palmitoyl transferase 1C (CPT1C), an enzyme located in the outer mitochondrial membrane, is involved in fatty acid transport and oxidation and in cell proliferation, a potential driver of cancer cell senescence. The study confirmed that CPT1C is a novel target gene of PPARα, and that knockout of PPARα leads to a decrease in CPT1C expression, which inhibits the proliferation of MDA-MB-1 and PANC-1 tumor cell lines in a CPT1C-dependent manner (Chen et al., 2017a). Mitochondrial 3-hydroxy-3-methylglutaryl-CoA synthase (HMGCS2) is a rate-limiting enzyme for ketogenesis that catalyzes the first enzymatic reaction in the ketogenic process, and HMGCS2 expression is associated with clinical prognosis and poor prognosis in patients with colorectal cancer and oral squamous cell carcinoma. Studies have demonstrated that PPARα and HMGCS2 directly interact, resulting in the activation of the proto-oncogene Src and promoting the growth and invasion of malignant tumors (Saraon et al., 2013; Chen et al., 2017b). PPARα signaling also improves lipid turnover, which is the rate at which lipids are removed and stored in fat cells, and maintains the high energy requirements of tumor cells to ensure the stemness and self-renewal ability of pancreatic and colorectal cancer stem cells (Kuramoto et al., 2021). Given the interaction between PPAR-α and hormone metabolism, the researchers found that activated PPAR-α increased the expression and activity of CYP1B1 (a subtype of cytochrome P450), further influencing the occurrence and progression of hormone-dependent tumors including breast cancer through the biotransformation of endogenous estrogens and environmental carcinogens (Hwang et al., 2019).

In terms of cancer suppression, studies have demonstrated that, on the one hand, PPARα limits the process of mitochondrial oxidative phosphorylation (OXPHOS), and non-tumor cells are not affected by it; on the other hand, PPARα increases the production of ROS, leading to the accumulation of mitochondrial oxidative stress in melanoma cells. At the same time, the activation of PPARα also promotes the amp-activated protein kinase (AMPK) signaling pathway, which increases the oxidation of fatty acids, while effectively inhibiting the glycolysis of tumor cells, further reducing the production of ATP and resisting the occurrence of oral cancer (Chang and Huang, 2019; Grabacka et al., 2020). In addition, PPARα in HCT-1, SW1, HeLa, and MCF-116 cancer cell lines reduced levels of the Glut480 (glucose transporter 7) protein. In contrast, silent PPARα reversed this phenomenon. On further analysis, PPARα directly targeted the consensus PPRE motif in the Glut1 promoter region, inhibiting Glut1 transcription, which in turn led to a decrease in glucose inflow in cancer cells (You et al., 2017). The lipid-lowering drug fenofibrate as a PPARα agonist induces metabolic reprogramming in oral cancer, changing the protein expression of hexokinase II (HK II), pyruvate kinase, pyruvate dehydrogenase, and voltage-dependent anion channel, thus delaying tumor development (Jan et al., 2016). In addition, fenofibrate exhibits antitumor activity *in vitro* and *in vivo* through mitochondrial and metabolic reprogramming, altering glucose and lipid metabolism, inhibiting the proliferation of gastric cancer cells, and promoting apoptosis of gastric cancer cells, indicating mitochondrial regulation and normalization of cancer cell metabolism as new therapeutic strategies for cancer (Chen et al., 2020).

### 3.2 PPARγ

Similarly, although PPARγ has been extensively studied in the metabolic regulation of tumor cells, due to its complex regulatory network does not allow conclusions to be reduced to simple cancer promotion or suppression. A study in 2009 reported that breast cancer cells positive for ERBB2 (an epidermal growth factor receptor, a marker of poor prognosis) produce large amounts of fat due to the activation of PPARγ, a key pathway for these cells to produce energy and survive. Activated PPARγ enables ERBB2-positive breast cancer cells that produce high levels of fat to convert fatty acids into triglycerides, allowing these cells to avoid cell death caused by lipotoxicity (Kourtidis et al., 2009). After PPARγ was identified as a mature positive regulator of adipogenesis and lipid storage in 2013, inhibition of PPARγ was reported to reduce aldehyde dehydrogenase (ALDH) activity in ERBB2-positive breast cancer cells. The results of an *in vitro* tumor spheroidization assay showed that the PPARγ antagonists GW9662 and T0070907 reduced tumor spheroids formation in ERBB2-positive cells (Wang et al., 2013). A 2014 study showed that activated PPARγ inhibited lung cancer cell proliferation by metabolic changes. Treatment with PPARγ agonist pioglitazone triggers metabolic switches that inhibit pyruvate oxidation and reduce glutathione levels, and these metabolic changes lead to a significant increase in ROS levels and cell cycle arrest (Srivastava et al., 2014). This was followed in 2015 by the discovery of a link between the induction of PPARγ activity and concomitant autophagy cell death in chronic myeloid leukemia cell lines (K562 and KCL-22). The anticancer fatty acid derivative AIC-47 binds and activates PPARγ, indirectly reduces the expression level of oncogene c-Myc, and leads to β-catenin inactivation and increase of the PKM1/PKM2 ratio. Metabolism shifts from glycolysis to the tricarboxylic acid cycle, and at the same time, ROS levels increase, inducing autophagy in cancer cells (Shinohara et al., 2015). The following year, a study based on Sleeping Beauty confirmed that the insertion of mutations in the PPARγ gene made mice more tumor-aggressive. PPARγ overexpression determines the upregulation of enzymes involved in *de novo* fatty acid synthesis, and in contrast, this effect is masked by PPARγ knockout (Ahmad et al., 2016). Similarly, in prostate cancer, overexpression of PPARγ promotes Akt3 activity, inhibits nuclear output protein CRM1, and enhances nuclear retention of PPARγ coactivator 1α (PGC1α). This activity increases mitochondrial ATP output in cancer cells to meet the high energy demands of EMT and cancer cell metastasis (Galbraith et al., 2021).

In our cancer suppression review, we reported that PPARγ stimulates adipogenesis in colorectal and breast cancer cells, disrupting the YAP-Hippo signaling pathway, thereby forcing terminal differentiation and inhibiting the proliferation of cancer cells (Sarraf et al., 1998; Basu-Roy et al., 2016). Ornithine decarboxylase 1 (ODC1) is a metabolic enzyme key involved in polyamine biosynthesis, typically upregulated in hepatocellular carcinoma. After siRNA silenced ODC1, the downregulation of ODC1 led to the upregulation of Krüppel-like factor 2 (KLF2), which in turn led to a decrease in PPARγ levels, inhibition of the expression of important regulators affecting glucose transport and lipid biogenesis in cancer cells, and a significant reduction in lipid droplet accumulation (Banerjee et al., 2003; Choi et al., 2016).

In addition, we found that several typical epithelial cancers share a common feature: disruption of the Wnt/β-catenin pathway, which often leads to the upregulation of enzymes associated with aerobic glycolysis. In many tissues, PPARγ activation induces inhibition of the β-catenin pathway, and activation of the typical Wnt/β-catenin pathway inactivates PPARγ. The development of most cancers is often accompanied by downregulation of PPARγ and upregulation of Wnt/β-catenin pathway (Lecarpentier et al., 2017). Specifically, ligand-activated Wnt ligands trigger nuclear translocations of β-catenin and bind to target genes, including pyruvate dehydrogenase kinase (PDK), monocarboxylic lactate transporter-1 (MCT-1), c-Myc, and COX-2, while downregulation of PPARγ is associated with upregulation of Wnt/β-catenin. PDK1 acts as phosphorylating pyruvate dehydrogenase, which is converted to lactate by activating lactate dehydrogenase. Simultansously, MCT-1 is involved in the secretion of extra cytoplasmic lactic acid. Therefore, PPARγ inhibits PDK1 and MCT-1 gene transcription, resulting in ineffective activation of the Wnt/β-catenin pathway (Abbot et al., 2005; Lecarpentier et al., 2014). Activated PPARγ promotes cell cycle arrest, cell differentiation, and apoptosis, while downregulation of the Wnt/β-catenin pathway reduces the release of inflammatory factors (TNF-α, TGF-β, IL-6, and IL-8) and oxidative stress. This pathway inhibits hepatocellular carcinoma metastasis (Vallée and Lecarpentier, 2018; Zuo et al., 2021).

### 3.3 PPARβ/δ

Compared with that of the other two PPARs, the cancer-promoting effect of PPARβ/δ is more widely recognized. Early studies have shown that PPARβ/δ is a transcription factor associated with metabolic gene regulation and inflammation, which is related to tumor promotion and PDK1 regulation, as a key regulator of the AGC protein kinase family, which includes proto-oncogenes such as Akt and PKB associated with several malignancies, including breast cancer. The PI3K/Akt pathway is known to phosphorylate and activate ATP citrate lyase, the target gene Pdk4 of PPARβ/δ slows the flow of pyruvate to oxidative phosphorylation, and Acss2 (a member of the acyl-CoA synthase short-chain family 2) promotes the conversion of lactate to pyruvate. These three proteins work synergistically to increase the content of acetyl-CoA to promote glycolysis and fatty acid synthesis, while the PPARβ/δ agonist GW501516 improves their prometabolic efficiency and increases cancer cell aggressiveness in cancer model mice (Di-Poï et al., 2002; Pearce et al., 2010; Pollock et al., 2011).

To explore the mechanism whereby PPARβ/δ expression in patients with breast cancer is inversely correlated with survival, human breast cancer cells were cultured under conditions of low glucose and other endoplasmic reticulum stress (such as hypoxia), and cells overexpressing PPARβ/δ had the better potential for multiplication. Conversely, cells that knocked down PPARβ/δ expression had a proliferation rate comparable to that of the control group, and this was associated with elevated levels of catalase and Akt protein. In summary, PPARβ/δ regulates the viability of breast cancer cells in harsh environments by reducing oxidative stress and enhancing metabolic efficiency (Wang et al., 2016). A similar phenomenon has been found in chronic lymphocytic leukemia (CLL) cells: glucocorticoids or synthetic PPARβ/δ agonists are upregulated in depleted tissue culture media (low glucose levels, hypoxia, and exposure to cytotoxic drugs), protecting CLL cells from metabolic stressors (Li et al., 2017). Follow-up studies demonstrated that lipid-activated PPARβ/δ in CLL induces high cholesterol and plasma membrane levels and enhances interferon-dependent STAT phosphorylation (Sun et al., 2018). To further investigate the effect of PPARβ/δ on the survival of cancer cells in harsh environments, Jeong et al. found that the expression of hypoxia-induced tumor-promoting cytokines IL-2 and VEGF was significantly weakened in PPARδ-deficient HCT116 colon cancer cells; in other words, PPARδ deletion led to colon cancer cells failing to stimulate endothelial cell angiogenesis and macrophage migration. PPARβ/δ is regulated by PI3K/Akt upstream, but can itself regulate the expression of PI3K and Akt; that is, there may be a closed-loop system between the two (Jeong et al., 2014).

Based on the upstream regulation of promyelocytic leukemia (PML) protein, a PML-PPARβ/δ-fatty acid oxidation pathway maintained hematopoietic stem cell (HSC) activity, and the activation of PPARβ/δ increased the asymmetric cell division of HSCs, which has potential therapeutic significance (Ito et al., 2012). Later, PML was found in breast cancer as a negative regulator of the acetylation of PPARs co-activator 1 A (PGC1A), and an effective activator of PPARs signaling and fatty acid oxidation (Carracedo et al., 2012). Based on the ability of transmembrane transporters to transport nutrients from the extracellular compartment to the cytoplasm, K-Ras-mediated glucose transporter-1 (Glut1) expression leads to increased glucose uptake and cell survival under low glucose conditions, and PPARδ directly regulates Glut1 gene transcription, increasing glucose and amino acid uptake, activating mTOR signaling, resulting in tumor progression. Conversely, silencing PPARδ inhibited this process, and in addition, PPARδ promoted chemoresistance in cancer cells, which was alleviated by PPARδ antagonists (Ying et al., 2012; Boroughs and DeBerardinis, 2015; Zhang et al., 2017). Another study reached similar conclusions, where activating PPARδ significantly increased Glut1 and solute carrier family member 5 (SLC1A5) gene and protein expression in multiple cancer cell lines (HCT-1, SW1, HeLa, and MCF-1), while metformin inhibited this, which was associated with metformin-mediated inhibition of PPARδ activity (Ding et al., 2019).

Long-chain fatty acids (LCFAs) are energy sources, building blocks of cell membranes, and constituent precursors of signaling molecules. Dietary fatty acids are associated with colon cancer risk, saturated long-chain fatty acids (SLCFAs) are positively correlated with colon cancer risk, and unsaturated long-chain fatty acids (ULCFAs) are negatively correlated. A recent study linking LCFA to PPARβ/δ showed that LCFA binds FABP5 from the cytoplasm to nuclear PPARβ/δ and replaces endogenous ligands and retinoic acid conduction, where SLCFAs inhibit the FABP5-PPARβ/δ pathway to inhibit cancer and ULCFAs activate the FABP5-PPARβ/δ pathway to increase cancer cell proliferation (Hardy et al., 2000; Li et al., 2011; Hodge et al., 2015; Levi et al., 2015).

## 4 Advances in targeting PPARs in cancer therapy

Due to the potent metabolic regulatory properties and gene targeting of PPARs, PPAR regulators have been widely used in the treatment of several diseases, including obesity, dyslipidemia, type II diabetes, and various metabolic disorders. Strong evidence suggests that PPAR modulators regulate cancer cell proliferation and differentiation, but the results are sometimes unclear or even contradictory, and the relevant progression of each of the three PPAR subtypes in cancer is described below (Heudobler et al., 2018; Cheng et al., 2019) (Table 1–3).

**TABLE 1:** Summary of the application of PPARα-targeting drugs in cancer therapy.

Medicine	Manner	Cancer type	Experimental model	Mechanism
**Fenofibrate**	Excitation	Mantle cell lymphoma	MCL cell line	Downregulate the survival gene TNF-α, reduces the nuclear translocation of NFκB
	Excitation	Breast cancer	TNBC cell line	Activate the NF-κB pathway to induce apoptosis of breast cancer cells
	Excitation	Melanoma, breast cancer, Lewis lung cancer	B16-F10, MDA436, LLC cell line	Inhibit endothelial cell proliferation and VEGF production
	Excitation	Lymphoma	C57Bl/6J and PPARα knock-out mice	Stimulate the liver to the intake of free fatty acids, restore the liver fatty acid oxidation capacity
	Excitation	Liver cancer	Huh7 human hepatoma cell line	Inhibit the proliferation of Huh7 cells by blocking Akt activation
	Excitation	Glioma	LN-229 and T98G glioma cell line	Inhibit IGF-IR and lead to ROS accumulation
	Excitation	Oral cancer	Cal27 human oral cancer cell line	Trigger AMPK pathway inhibits Akt, downregulates mTOR activity
	Excitation	Glioblastoma	U87 and U251 glioblastoma cell line	Impair mitochondrial respiration
	Excitation	Gastric cancer	MGC803 and SGC7901 human gastric cancer cell line	Induce mitochondrial reprogramming
**Clofibrate**	Excitation	Breast cancer	SUM149PT and SUM1315MO2 breast cancer cell line	Led to cell cycle arrest and inducing the level of pro-apoptotic factor P21
	Excitation	Liver cancer	HepG2 human liver cancer cell line	Induce cell apoptosis
	Excitation	Colorectal cancer	SW480 colorectal cancer cell line	Promote autophagy of tumor cells
	Excitation	Pancreatic cancer	Several pancreatic cancer cell line	Downregulate the expression of PTPRZ1 and Wnt8a
**Wy14643**	Excitation	Colorectal cancer	Mouse model of DSS induced colitis	Reduced levels of inflammatory cytokines
	Excitation	Non-small cell lung cancer	Wild type and CYP2C44-NULL (Cyp2c44 KO) mice	Downregulate Cyp2c44 expression and circulating EET to inhibit tumor angiogenesis
	Excitation	Colon cancer	SW620 colon carcinoma cell line	Reduce the transcriptional activation of genes involved in inflammatory response
	Excitation	Colorectal cancer	Several colorectal cancer cell line	Inhibits Glut1 transcriptional activity, glucose uptake, and mTOR pathways
**GW6471**	Inhibition	Breast cancer	MDA-MB-231 Breast cancer cell line	Reduce the viability, proliferation and globular formation of cancer stem cells
	Inhibition	Renal cell carcinoma	VHL (+) and VHL (−) RCC cell line (786-O and Caki-1)	Induce apoptosis and cell cycle arrest at G1/G4
	Inhibition	Head and neck paraganglioma	Primary HNPGL cells from HNPGL patients	Inhibit PI3K/GSK3β/β-catenin signaling pathway, induce cell apoptosis
**NXT629**	Inhibition	Chronic lymphocytic leukemia	CLL cells taken from patients and CLL mouse model	Induce CLL cell apoptosis and reduce the tumor burden of CLL cell proliferation *in vivo*
**AA452**	Inhibition	Glioblastoma	GB patient-derived GB primary cell	Reduce intracellular cholesterol esters and lipid droplets, restrict cancer cell proliferation
**MK866**	Inhibition	Chronic lymphocytic leukemia	CLL cells taken from patients	Kill the circulating CLL cells

MCL: mantle cell lymphoma, TNBC: triple-negative breast cancer, EET: epoxyeicosatrienoic acids, Cyp2c44: cytochrome P450 arachidonic acid epoxygenases; Bad: Bcl-2-associated death protein; VEGF: vascular endothelial growth factor; AMPK: adenosine 5‘-monophosphate -activated protein kinase; ERK: extracellular regulated protein kinases; Bcl2: B-cell lymphoma-2; PTPRZ1: protein tyrosine phosphatase receptor type Z1; Wnt8a: Wnt family member 8A; HNPGL: head and neck paraganglioma; GB: glioblastoma; CLL**:** chronic lymphocytic leukemia.

**TABLE 2:** Summary of the application of PPARγ-targeting drugs (Excitation) in cancer therapy.

Targeted medicine	Cancer type	Experimental model	Mechanism
**Ciglitazone**	Prostate cancer	p53+/+ and P53^−/−^ prostate cancer cell	Inhibit the anti-apoptotic function of Bcl-xL and Bcl-2
	Ovarian cancer	Ovarian cancer cell line	Block the TNF family death receptor signaling pathway
	Brain cancer	T98G and DB29 glioma cell line	Inhibit the proliferation of BTSC
	Breast cancer	MCF-1 breast cancer cell line	Inhibit cyclin D1 and expression of estrogen receptor alpha
	Ovarian cancer	Ovarian cancer implantation mouse model	Decrease the expression of COX-2, mPGES, and EP2, result in decreased angiogenesis and apoptosis
**Troglitazone**	Breast cancer	MCF-7 breast cancer cell line	Downregulate MMP-9 inhibited MCF-7 cell invasion
	Prostate cancer	PC-3 prostate cancer cell line	Upregulate E-cadherin and GPx3, reduce the growth and invasion of cancer cells
	Colon cancer	Colorectal cancer cell line	Modulate E-cadherin/beta-catenin pathway and Promote cancer cell differentiation
	Thyroid cancer	TPC-1, FTC-133, FTC-236, FTC-238, XTC-1 and ARO82-1 cell line	Downregulate CD133 surface expression and upregulate NIS, induce ant proliferation and redifferentiation of thyroid cancer cell lines
	Breast cancer	MDA-MB-75 and ZR-1-2 cell lines	Upregulate apoptosis signaling pathway
	Breast cancer	MCF-7, BT20, BT474 and other breast cancer cell line	Combined with retinoid receptors, inhibit the proliferation of cancer cells
	Gastric cancer	SGC7901 gastric cancer cell line	In combination with RXR agonists, increased Bax/Bcl-7901 levels were induced to inhibit SGC2 cell proliferation
	MPM	MPM cell line	Induce G1 cell cycle arrest and apoptosis
	Ovarian cancer	HEY cell line	Combined TRAIL overcomes chemical resistance in ovarian cancer
	Cervical cancer	HeLa, Ca Ski, C-33 A cell line	Decreased E6 virus oncoprotein expression, restore TRAIL sensitivity
	Breast cancer	MDA-MB-7 and MCF-7 cell line	Combined with tamoxifen to inhibit cancer cell proliferation
	Lung cancer	CL1-1 and A0 cell line	Downregulated Cdk2, E2F-1 and cyclin B1 to induce cell apoptosis
	thyroid cancer	Mouse xenotransplantation model	Result in G0/G1 phase cell cycle arrest
**Pioglitazone**	Glioma	Human glioma cell line	Inhibit glioma cell growth and invasion based on β-catenin
	Breast cancer	Human breast cancer cell line	Inhibit expressions of estrogen receptor and aromatase
	Liver cancer	From human primary cancer cell	Block the RAGE signaling to inhibit the growth and invasion of cells
	Lung cancer	NCI-H2347 and NCI-H1993 cell line	Inhibit pyruvate oxidation and increase ROS level
**Rosiglitazone**	Melanoma	A375 Human melanoma cancer cell line	Inhibit ERK phosphorylation and inhibit MAPK/ERK pathway
	Prostate cancer	PC-3 human prostate carcinoma cell	Inhibit CXCL12/CXCR4 axis, downregulate CxCl12-induced migration and invasion
	Pancreatic cancer	SW1990 pancreatic cancer cell line	Upregulate Bax, inhibit COX-2 and induce apoptosis of pancreatic cancer cells
	Breast cancer	MCF-7, MDA-MB-231 and T47D human breast cancer cells	Bind and inhibit NHE1, lead to sensitization of tumor cells to death
	Breast cancer	MCF-1 breast cancer cell line	Promote BRCA1 to induce cell apoptosis
	Adrenocortical carcinoma	SW13 adrenocortical cancer cells	Increase BECLIN-1 and LAMP-1 expression to promote autophagy
	Hepatocellular carcinoma	BEL-7402 and Huh-7 cell line	Promote the antitumor effect of 5-FU
**Efatutazone**	Lung adenocarcinoma	Lung adenocarcinoma cell line	Upregulate phosphatase and PTEN and inactivation of Akt pathway
	Lung adenocarcinoma	HCC827-GR and PC9-GR cell lines	Inhibit the expression of LXR-α and ABCA mRNA, and inhibit the proliferation
	Breast cancer	DCIS, MCFDCIS cells	Downregulate Akt phosphorylation and reduce tumor ball formation
**Balaglitazone**	Leukemia	K562/DOX human myeloid leukemia cells	Upregulate PTEN expression and reversal of drug resistance
**CB13**	Non-small cell lung cancer	3T3-L1, A549 and H460 cell line	Induce endoplasmic reticulum stress and cell death

BTSC: brain tumor stem cells; GPx3: glutathione peroxidase 3; NIS: sodium iodide cotransporter; CD133: cluster of differentiation 133; ERK: extracellular regulated protein kinases; MAPK: mitogen-activated protein kinase; NHE1: Na^+^/H^+^ transporter gene; BRCA1: Breast cancer susceptibility gene 1; LAMP-1: Lysosomal Associated Membrane Protein 1; PTEN**:** phosphatase gene; LXR-α: liver X receptor alpha; ABCA: ATP-binding cassette transporter A1; PERK:PKR-like ER, kinase; CHOP:C/Ebp-Homologous Protein; ATF4: activating transcription factor 4; mPGES: microsomal PG E synthase; RXR: retinoid receptor; MPM: malignant pleural mesothelioma; TRAIL: tumor necrosis factor-associated apoptosis-inducing ligand.

**TABLE 3:** Summary of the application of PPARβ/δ-targeting drugs in cancer therapy.

Targeted medicine	Cancer type	Experimental model	Mechanism
**GW0742**	Breast cancer	MDA-MB-231and MCF7 cell line	Inhibit proliferation without promoting apoptosis
	Colorectal cancer	DSS induced mouse model	Remit colorectal cancer progression caused by colitis
	Liver cancer	HBV mouse model	Inhibit steatosis and cell proliferation, enhance hepatocyte apoptosis and regulate anti-inflammatory activity in Kupffer cells
	Colon cancer	PPARβ^+/+^ (−/−) mouse model	Reduce chemically-induced colon cancer and reduce intestinal polyps
	Melanoma	A0 and B10F100 cell line	Downregulate the expression of WT1 and inhibit the proliferation of melanoma cells
	Breast cancer	MCF0742 cell line	Upregulate ANGPTL4 to inhibit the proliferation of cancer cells
	Melanoma	UACC501516 cell line	Upregulate ANGPTL4 to inhibit the proliferation of cancer cells
	Melanoma	Melanoma cell lines	Inhibit melanoma cell proliferation by inhibition of WT1
	Melanoma	UACC903 cells lines	Upregulate ANGPTL4 to inhibit the spread of cancer cells
**L165041**	Lung cancer	A549 lung cancer cell line	Control negative growth of lung cancer cells based on PGI2 signal
**GW501560**	Breast cancer	MCF-7, MDA-MB-231 breast cancer cell lines	The interaction with c-Myc inhibits tumorigenicity of breast cancer cells
**Antagonist GSK3787**	Colorectal cancer	Human colorectal cancer organoid cells	Downregulate connexin 43, PDGFRβ, AKT1, EIF4G1, and CDK1 to inhibited proliferation

WT1L: wilms tumor inhibitor; ANGPTL4: angiopoietin-like protein 4; PGI2: prostaglandin I2; PDGFRβ: platelet derived growth factor receptor beta; AKT1: AKT, serine/threonine kinase 1; EIF4G1: eukaryotic initiation factor G1; CDK1: Cyclin-dependent kinase 1; WT: Wilms tumor suppressor 1; ANGPTL4: Angiopoietin-like protein 4.

### 4.1 PPARα

The synthetic ligands of PPARα include fenofibrate, clofibrate, and wyeth14643, which are not only particularly effective clinical lipid-lowering drugs but also effective anticancer drugs. Like other fibrates, fenofibrate is mainly used to reduce cholesterol levels in patients at risk of cardiovascular disease, and its anti-cancer effects have recently been reported: 1) Inhibition of B-cell lymphoma in mice by regulation of lipid metabolism, specifically, stimulation of the uptake of free fatty acids in the liver, and restoration of the oxidation ability of liver fatty acids, thereby accelerating the clearance of lipids released by white adipose tissue (Huang et al., 2013). 2) Fenofibrate-mediated IGF-IR inhibition with PPARα-dependent metabolic induction and the resulting accumulation of ROS helps counteract glioma cell spread; likewise, fenofibrate-induced PPARα activation inhibits IGF-I-mediated growth and survival responses of medulloblastoma cell lines (Urbanska et al., 2008; Drukala et al., 2010). 3) based on the weakening of MMPs expression, the enhancement of AMPK phosphorylation, and the inhibition of NF-κB and its DNA-binding activity, fenofibrate resists the invasion and migration activity of Cal27 human oral cancer cells; in addition, fenofibrate activation of PPAR-α may induce a decrease in the migration ability of oral cancer cells *in vitro* by interfering with the Warburg effect, and the mechanism may be to trigger the AMPK signaling pathway to inhibit Akt, downregulate mTOR activity through tsc1/2-dependent signaling pathway, and regulate the mitochondrial oxidative phosphorylation Warburg effect to control the energy-generating pathway (Tsai et al., 2016). 4) Fenofibrate not only alleviates glycolysis and lactic acid production in glioblastoma cells, but also damages the mitochondrial respiration of glioblastoma cells by inhibiting the transcriptional activity of NF-κB/RelA and destroying its association with hypoxia-inducible factor 1α (Han et al., 2015). 5) In gastric cancer, fenofibrate induces mitochondrial body mass programming through CPT1 and fatty acid oxidation pathways, as well as activating the AMPK pathway and inhibiting the HK2 pathway, thereby regulating glycolipid metabolism, inhibiting the growth of gastric cancer cells, and causing apoptosis of gastric cancer cells (Chen et al., 2020).

The combination of fenofibrate and tretinoin is a potent inhibitor of the growth of endometrial cancer cells *in vitro*; that of budesonide and fenofibrate has a significant inhibitory effect on A549 lung cancer cells, and its mechanism is related to G1 cell cycle arrest, NF-κB activity, and ERK signaling pathway. When docetaxel/mitoxantrone is combined with fenofibrate in prostate cancer cell lines, it increases the chemosensitivity of prostate cancer cells by interfering with energy metabolism and damaging drug-resistant cells (Saidi et al., 2006; Liang et al., 2014; Luty et al., 2019).

Clofibrate is a fibroic acid derivative that is clinically used as a lipid-lowering drug; in addition, it shows significant cytotoxicity to breast cancer cells. Specifically, clofibrate inhibits the growth of breast cancer cells by inhibiting the activation of NF-κB and extracellular regulatory protein kinase 1/2 (ERK1/2), inhibiting the cyclin factor cyclin D1, cyclin A, and cyclin E, leading to cell cycle arrest and inducing pro-apoptotic factor P21 levels (Rakhshandehroo et al., 2010; Chandran et al., 2016). Clofibrate also causes apoptosis in human liver cancer HepG2 cells in a time- and concentration-dependent manner by increasing the expression of protein phosphatase-2A and Bcl-2 pro-apoptotic factor family BAD (Maggiora et al., 2010). For colorectal cancer SW480 cells, clofibrate significantly inhibits tumor proliferation and sensitizes SW480 cells to chemotherapy drugs in a PPARα-dependent manner, thereby inducing anti-apoptotic Bcl2 protein degradation and promoting autophagy in tumor cells (You et al., 2018). PPARα activated by clofibrate regulates cell cycle progression and apoptosis in pancreatic cancer cell lines, and the expression of PTPRZ1 and Wnt8a, two core components of the β-catenin pathway, is downregulated by clofibrate, increasing the sensitivity of pancreatic cancer cells to radiation therapy (Xue et al., 2018).

Wy14643 is a commonly used PPARα agonist in breast cancer. In a DSS-induced mouse model of innate immune-mediated colitis, Wy-14643-activated PPARα inhibited colorectal cancer by reducing inflammatory factor levels (Azuma et al., 2010). WY14643 attenuates the early stages of colon tumorigenesis by reducing AP-1 (activator-1)-mediated transcriptional activation of genes involved in the inflammatory response such as Cox-2 and VEGF in a PPARα-dependent manner (Grau et al., 2006). In addition, WY14643 can increase chemosensitivity by affecting the transcriptional activity of glucose transporter-1, inhibit the mTOR pathway, and lead to apoptosis of cancer cells (Gou et al., 2019). Wy14643 combined with Benzafibrate also significantly inhibited lung cancer cell growth by activating PPARα (Skrypnyk et al., 2014).

Notably, in recent years, the research on synthetic PPARα antagonists has considerably increased. For example, the antagonist GW6471 has anti-breast cancer cell proliferation and apoptotic effects. It can also induce apoptosis and cell cycle arrest of renal cell cancer cells and inhibit the process of glycolysis, hindering the progression of renal cell carcinoma. Based on the PI3K/GSK3β/β-catenin pathway, GW6471 is associated with decreased viability and proliferation of head and neck paraganglioma (HNPGL) cells, which interferes with the cell cycle and induces apoptosis, thereby inhibiting the proliferation and migration of HNPGL cells (Abu Aboud et al., 2013; Florio et al., 2017; Castelli et al., 2021). The antagonist AA452 elicited metabolic reprogramming within tumor tissue and was found to increase sensitivity to radiotherapy in human glioblastoma primary cells by reducing intracellular cholesterol esters and lipid droplets while regulating the mevalonate pathway, thereby limiting cancer cell proliferation and migration (Benedetti et al., 2017).

### 4.2 PPARγ

Thiazolidinedione drugs (TZDs) are a class of synthetic ligands for the synthesis of PPARγ, including rosiglitazone (ROSI), pioglitazone (PGZ), troglitazone (TGZ), and ciglitazone, which are commonly used clinically to lower blood sugar and resist cardiovascular and cerebrovascular diseases. In anti-cancer therapy, this class of drugs is involved in the cell cycle, apoptosis, and hormonal response, and other aspects of regulation, including intracellular Ca2+ depletion, proteasome degradation to induce cell cycle arrest and transcriptional inhibition of related hormone receptors, and reduction of macrophage activation, while they also induce apoptosis to inhibit cancer cell proliferation by participating in reducing the expression of c-Myc, Bcl2, VEGF, and b-FGF (Wei et al., 2009; Zhang et al., 2013; Fröhlich and Wahl, 2015).

Initially, ciglitazone inhibits the cell cycle of cancer cells by partially consuming Ca2+ in cells, resulting in the passivation of eukaryotic initiation factor 2 to inhibit translational initiation and exert anticancer activity (Palakurthi et al., 2001). In the apoptotic pathway, ciglitazone can inhibit the anti-apoptotic function of Bcl-xL and Bcl-2 and improve the abnormal intrinsic apoptotic activity of prostate cancer cells (Shiau et al., 2005). By selectively inhibiting the apoptosis inhibitor protein FLIP, blocking early events of the TNF family death receptor signaling pathway, ciglitazone downregulates apoptosis signaling to eliminate tumor cells (Kim et al., 2002). Ciglitazone and another PPARgamma agonist 15d-PGJ2 inhibit the viability and proliferation of brain cancer stem cells by inhibiting SOX2 while enhancing the expression of the NANOG gene (Pestereva et al., 2012).

PGZ-activated PPARγ targets estrogen receptors (ER) and aromatase, activates the tumor suppressor gene PTEN to inhibit ER expression or induce proteasome-dependent ER degradation, and inhibits aromatase through PGE2 and BRCA1 signaling pathways to prevent breast cancer progression (Margalit et al., 2012). Advanced glycosylated end product (RAGE) receptor is significantly expressed in human hepatocellular carcinoma (HCC), and is closely related to the pathological stage and tumor invasion. After PGZ treatment, PPARγ expression in hepatoma cells was elevated and the growth and invasion of HCC cells were inhibited by blocking RAGE signaling (Yang et al., 2015). According to Srivastava et al., PGZ activation of PPARγ induces metabolic switches; PPARγ inhibits pyruvate oxidation by inhibiting pyruvate dehydrogenase kinase 4 (PDK4) or β-oxidation of fatty acids and reducing glutathione levels, resulting in a significant increase in ROS levels and ultimately inducing cell cycle progression in lung cancer cells (Srivastava et al., 2014).

ROSI is a second-generation thiazolidinedione PPARγ agonist originally developed as an insulin sensitizer for the treatment of diabetes (Chi et al., 2021). The PI3K/AKT/mTOR and MAPK/ERK pathways are two important cell proliferation signaling pathways, among which PTEN is a natural inhibitor of the PI3K/AKT pathway, and ROSI activates PPARγ in HCC cell lines to bind to PTEN promoters and increase the expression of PTEN. MAPK/ERK is a signaling pathway from membrane receptors to the nucleus, and PPARγ activated by ROSI will inhibit phosphorylation of ERK, thereby inhibiting the growth of melanoma and breast cancer cells (Liu et al., 2006; Moon et al., 2010). In terms of cancer metastasis, ROSI activates PPARγ and inhibits the activation of the PI3K-Akt pathway by inhibiting downregulation of CXCL12-induced migration and invasion on the CXCL12/CXCR4 axis in prostate cancer cell lines. In addition to chemokines, ROSI also inhibits the expression of some migration-related genes, including MMP-7, COX-2, and TIMP-1, thereby reducing tumor metastasis (Miao et al., 2011; Qin et al., 2014). ROSI also plays a certain role in promoting the apoptosis of tumor cells. Overexpression of COX-2 may be native, and inhibition of COX-2 after ROSI activation of PPARγ has been shown to improve treatment outcomes in colon cancer. The Bcl-2 family is an effective regulator of apoptosis, while ROSI induces apoptosis in pancreatic cancer cells by upregulating the expression of the Bax acceleration factor (Sun et al., 2009). The Na+/H+ transporter gene NHE1 is a permeable homeostatic regulator. Decreases in its expression cause tumor cell sensitivity to death, its promoter region has a PPARγ response element, PPARγ can bind and inhibit its expression, and histopathological analysis of breast cancer biopsies obtained from patients with type II diabetes treated with ROSI has shown significant inhibition of NHE1 in tumor tissue (Kumar et al., 2009).

In addition, efatutazone is a novel PPARγ agonist belonging to the third generation of TZDs, and two independent studies reported that efatutazone upregulates PPARγ, phosphatase, and PTEN protein expression and inactivates the Akt pathway. This inhibits the EGFR-TKI resistance pathway in lung adenocarcinoma, which works synergistically with LXRα, a member of another class of nuclear receptors reported to be a potential target for the prevention and treatment of a variety of cancers (Ni et al., 2017; Ni et al., 2018). In a phase 1 trial, oral efatutazone enhanced the efficacy of paclitaxel in treating thyroid cancer (Smallridge et al., 2013). Balaglitazone, another member of the TZDs family, has shown elevated expression of PPARγ and PTEN, resulting in a partial reversal of P-glycoprotein-mediated multidrug resistance in doxorubicin-resistant human myeloid leukemia (K562/DOX) cells (Yousefi et al., 2017).

In terms of combined administration, the combination of ciglitazone and the chemotherapy drug cisplatin can improve the anti-cancer efficacy of human ovarian cancer (Yokoyama et al., 2011). Combined use of TGZ and human retinol X receptor RXR α ligand enhances the apoptosis and growth inhibitory effects on gastric and breast cancer cell lines (Elstner et al., 2002; Liu et al., 2013b). In ovarian and breast cancer cell lines that do not respond to conventional therapy, TGZ combined with the cell signaling molecule TNF-associated apoptosis-inducing ligand (TRAIL) showed effective synergistic pro-apoptosis results (Bräutigam et al., 2011; Plissonnier et al., 2017). Resistance to 5-fluorouracil (5-FU) is the main cause of chemotherapy failure in advanced hepatocellular carcinoma, and ROSI activation of PPARγ increases PTEN expression and decreases COX-2 expression, inducing sensitivity to 5-FU antitumor activity in HCC cell lines (Cao et al., 2009). In addition, other drugs including lovastatin, aspirin, and the estrogen modulator tamoxifen have been reported as synergistic antitumor drugs with TGZ against thyroid, glioblastoma, lung, breast, and cervical cancer (Yao et al., 2006; Yu et al., 2008; Yan et al., 2010; Zhong et al., 2018).

### 4.3 PPARβ/δ

As early as 1999, Kinzler et al. determined that PPARδ was the target of the tumor suppressor gene APC by analyzing the overall gene expression profile of human colorectal cancer (CRC) cells. PPARδ expression is elevated in CRC cells, and APC inhibits its expression by inhibiting β-catenin/Tcf-4 regulatory transcription. Given that nonsteroidal anti-inflammatory drugs (NSAIDs) can inhibit colorectal tumorigenesis and the ability of PPARs to bind to eicosasulfonic acids, PPARδ may also be a target for NSAIDs. APCs and NSAIDs inhibit the common target PPARδ, thus providing a link between the genetic alterations behind tumor development and cancer chemoprevention (He et al., 1999). Similarly, targeted inhibition of PPARβ/δ using aspirin in epithelial ovarian cancer cell lines expressing high levels of PPARβ/δ may reduce epithelial cell proliferation. Mechanistically, as a nonsteroidal anti-inflammatory drug that inhibits COX-1, aspirin impairs PPARβ/δ function and cell growth by inhibiting extracellular signal-regulatory kinase 1/2 (ERK1/2) (Daikoku et al., 2007).

In addition, several specific PPARβ/δ activators, GW0742, GW501516, L165041, and the antagonist GSK3787 play unique roles in cancer. The combination of GW0742 and the COX-2 inhibitor nimesulide can further reduce tumor diversity in wild-type mice (Zhu et al., 2010). In addition, GW0742 can slow the progression of colon cancer in mice and the carcinogenicity of human breast cancer cells, a phenomenon that was not observed in mice with PPARδ knockout (Ouyang et al., 2006; Yao et al., 2014). In transgenic hepatitis B virus mice, long-term treatment with GW0742 reduced the number of liver tumor foci, based on a decrease in cyclin D1 and c-Myc expression, and activated PPARδ reduced the proliferation of tumor cells (Balandaram et al., 2016). Another study showed that activation of PPARβ by GW0742 in mice led to increased expression of mRNA-encoding adipocyte differentiation-related proteins, fatty acid binding proteins, and cathepsin E, weakening chemically induced colon carcinogenesis (Marin et al., 2006). Culture of MCF0742 (breast cancer) and UACC501516 (melanoma) cells in the presence of GW0742 caused upregulation of the PPARβ/δ target gene angiopoietin-like protein 4 (ANGPTL4), and the growth of both tumor cell lines was inhibited (Girroir et al., 2008). GW0742-activated PPARβ/δ also inhibits the proliferation of different melanoma cell lines via its inhibition of Wilms tumor suppressor (WT1) promoter and direct transcriptional inhibition of its downstream target genes (Michiels et al., 2010). In addition, Liu et al. found that treatment with the PPARδ antagonist GSK3787 inhibited colorectal cancer tumorigenesis in mice (Liu et al., 2019a). Agonist L165041 induces apoptosis in lung cancer cell lines when used in combination with the nonsteroidal anti-inflammatory drug indomethacin (Fukumoto et al., 2005).

### 4.4 Carcinogenic side effects of PPARs-targeted drugs

Carcinogenic side effects of PPARγ agonists have been gradually discovered in recent years. To investigate the role of PPARs in diet-induced carcinogenesis, mice susceptible to intestinal tumors were treated with a synthetic PPARγ ligand. The mice developed multiple polyps in the colon, suggesting that PPARγ activation may provide a molecular link between a high-fat diet and an increased colorectal cancer risk (Saez et al., 1998). Similarly, animals treated with the PPARδ agonist GW501516 exhibited an accelerated formation of breast cancer tumors, particularly adenosquamous and squamous cell carcinomas, and tumors of mice treated with GW501516 exhibited increased levels of PPARδ and activated PDK1 (Yin et al., 2005). In a new transgenic mouse model, endogenous activation of PPARδ led to progressive histopathologic changes that resulted in estrogen receptor-positive, progesterone receptor-positive, and ErBB2-negative invasive ductal carcinoma. The incubation period of the mice was 12 months; however, the incubation period of the GW501516 treated mice was reduced to 5 months (Yuan et al., 2013). Another study showed that GW501560 increased VEGF expression in tumor cell lines in Apc (Min/+) mice, and VEGF directly promoted the survival of colorectal adenoma epithelial cells by activating PI3K-Akt signaling, which was manifested by a significant increase in the number and size of intestinal polyps (Gupta et al., 2004; Wang et al., 2006).

## 5 Conclusion

Cancer has long been considered the most harmful disease in the world due to its high morbidity and mortality rates. However, it is reassuring that many new therapeutic targets, such as PPARs, have demonstrated great potential in the inhibition of tumor proliferation and metastasis. Our study focused on the profound effects of PPARs on tumor tissue metabolism and TME. Previous studies have implicated PPARs in adipogenesis, lipid metabolism, insulin sensitivity, inflammation, and blood pressure regulation. PPARs-related metabolic disorders, such as obesity and type 2 diabetes, are independent risk factors for carcinogenesis and cancer prognosis predictors (Johnson et al., 2012; Hopkins et al., 2016; Mustafa et al., 2020). Although PPARs affect different aspects of TME and tumor cell metabolism, the three PPARs subtypes cannot be classified as having only pro- or anti-tumor effects because PPARs affect different cancers and cell types differently, and are perturbed by other cell signals. Notably, tumor cells are affected by PPARs while providing endogenous PPARs ligand signals to other cells in the TME, regulating PPARs activity through feedback, forming a more complex permanent closed loop. In conclusion, with the hope of providing valuable insights for better cancer treatment, we have attempted to establish the relationship between PPARs and cancer. However, the significance of PPARs-targeted drugs in cancer treatment has yet to be fully explored. The clinical applicability of PPARs-targeted drugs, the elimination of drug side effects, and the prognosis are the areas that require further research to gain satisfactory insights.

## Abbreviations

PPAR peroxisome proliferators-activated receptor; TME tumor microenvironment; WT1L wilms tumor inhibitor; ANGPTL4 angiopoietin-like protein 4; PPRE peroxisome proliferative response element; PGI2 prostaglandin I2; PDGFRβ platelet derived growth factor receptor beta; AKT1 AKT serine/threonine kinase 1; EIF4G1 eukaryotic initiation factor G1; CDK1 cyclin-dependent kinase 1; BTSC brain tumor stem cells; GPx3 glutathione peroxidase 3; NIS sodium iodide cotransporter; CD133 cluster of differentiation 133; ERK extracellular regulated protein kinases; MAPK mitogen-activated protein kinase; NHE1 Na+/H+ transporter gene; BRCA1 breast cancer susceptibility gene 1; LAMP-1 lysosomal associated membrane protein 1; PTEN phosphatase gene; LXR-α liver X receptor alpha; ABCA ATP-binding cassette transporterA1; PERK PKR-like ER kinase; CHOP C/Ebp-Homologous protein; ATF4 activating transcription factor 4; mPGES microsomal PGE synthase; RXR retinoid receptor; MPM malignant pleural mesothelioma; TRAIL tumor necrosis factor-associated apoptosis-inducing ligand; MCL mantle cell lymphoma; TNBC triple-negative breast cancer; PML Promyelocytic leukemia; EET epoxyeicosatrienoic acids; Cyp2c44 cytochrome P450 arachidonic acid epoxygenases; Bad Bcl-2-associated death protein; VEGF vascular endothelial growth factor; AMPK adenosine 5‘-monophosphate -activated protein kinase; ERK extracellular regulated protein kinases; Bcl2 B-cell lymphoma-2; VDAC Voltage-dependent anion channel; PTPRZ1 protein tyrosine phosphatase receptor type Z1; Wnt8a Wnt family member 8 A; HNPGL head and neck paraganglioma; GB glioblastoma; ROSI Rosiglitazone; PGZ Pioglitazone; TGZ Troglitazone; CLL chronic lymphocytic leukemia; HMGCS2 3-Hydroxy-3-Methylglutaryl-CoA Synthase 2; GLUT glucosetransportertype; FAS fatty acid synthase; CPTIC carnitine palmitoyltransferase 1C; OXPHOS oxidative phosphorylation; ALDH acetaldehyde dehydrogenase; FASN fatty Acid Synthase; ACLY ATP citrate lyase; Acss2 acetyl-coA synthetase 2; TSP-1 thrombospondin 1; Ang angiotensin; FGF2 fibroblast growth factor 2; PAI-1 plasminogen activator inhibitor 1
